# Coloured Noise from Stochastic Inflows in Reaction–Diffusion Systems

**DOI:** 10.1007/s11538-020-00719-w

**Published:** 2020-03-20

**Authors:** Michael F. Adamer, Heather A. Harrington, Eamonn A. Gaffney, Thomas E. Woolley

**Affiliations:** 1 Wolfson Centre for Mathematical Biology, Mathematical Institute, University of Oxford, Oxford, UK; 2 Cardiff School of Mathematics, Cardiff University, Cardiff, UK

**Keywords:** Coloured noise, Reaction–diffusion, Power spectra, Injectivity criterion, Chemical reaction networks

## Abstract

In this paper, we present a framework for investigating coloured noise in reaction–diffusion systems. We start by considering a deterministic reaction–diffusion equation and show how external forcing can cause temporally correlated or coloured noise. Here, the main source of external noise is considered to be fluctuations in the parameter values representing the inflow of particles to the system. First, we determine which reaction systems, driven by extrinsic noise, can admit only one steady state, so that effects, such as stochastic switching, are precluded from our analysis. To analyse the steady-state behaviour of reaction systems, even if the parameter values are changing, necessitates a parameter-free approach, which has been central to algebraic analysis in chemical reaction network theory. To identify suitable models, we use tools from real algebraic geometry that link the network structure to its dynamical properties. We then make a connection to internal noise models and show how power spectral methods can be used to predict stochastically driven patterns in systems with coloured noise. In simple cases, we show that the power spectrum of the coloured noise process and the power spectrum of the reaction–diffusion system modelled with white noise multiply to give the power spectrum of the coloured noise reaction–diffusion system.

## Introduction

One of the central challenges in mathematical biology is understanding mechanisms involved in development processes. Within the context of developmental biology, the emergence of large-scale spatial structure has often been theoretically explored through a common framework of deterministic partial differential equations defining reaction–diffusion systems (Murray 2008; Hochberg et al. 2003; Turing 1952). Whilst current frameworks may explain a variety of phenomena in development, they can also suffer from over-simplification (McKane et al. 2014), with the additional caveat that finding theoretical models both consistent with mechanism-based knowledge and capable of predicting observed patterns is a highly complex task, suffering from both model and parameter fine-tuning (Butler and Goldenfeld 2011; Xavier 2013).

Many models that describe pattern formation assume parameters are constant; however, this deterministic assumption is not suitable for certain conditions. Some systems are better suited towards a stochastic approach. When a system is coupled to external and stochastic drivers, the parameter values can change. The stochastic driving is often represented by stochastic parameters, that is a parameter which is drawn from a certain distribution at each time step or spatial point, and referred to as *extrinsic noise* below. This contrasts with most previous work on stochastic pattern formation, referred to as *intrinsic noise*, which assumes low copy number, and it does not assume external drivers as the source of noise (Schumacher et al. 2013; McKane and Newman 2005; McKane et al. 2014; Biancalani et al. 2011; Woolley et al. 2011a, 2012). Extrinsic noise has been studied extensively in García-Ojalvo and Sancho (2012), however, not in the context of chemical reaction network theory and reaction–diffusion systems.

The structure of intrinsic noise is often taken to be highly constrained and in particular uncorrelated in time, leading to white noise representations. For instance, if the noise is due to low copy number-induced dynamics, Gaussian white noise forcing emerges from the chemical Langevin equation approximation to the chemical master equation (Gillespie 2000). Even with such constraints, there is a rich diversity of noise-induced phenomena, such as spatio-temporal pattern formation (Schumacher et al. 2013; Woolley et al. 2012), stochastic oscillations (McKane and Newman 2005), metastability (McKane et al. 2014), waves (Biancalani et al. 2011) or enhanced oscillation amplitude (Dauxois et al. 2009), and a general introduction to the effects of noise in spatial system can be found in Sagués et al. (2007).

However, when the source of the noise is *extrinsic*, other forms of noise are permissible. In particular, the defining special properties of white noise may in general be relaxed and hence stochastic forcing can emerge with non-trivial temporal correlations, often termed *coloured noise*. Our objective is to show how extrinsic noise influences spatio-temporal reaction–diffusion patterns, in particular by developing power spectral methods to analyse the impact of coloured noise. In this paper, it is assumed that the effects of intrinsic noise are negligible and only the extrinsic noise provides a stochastic perturbation of the system. Therefore, in contrast to internal noise models, the common description of a stochastic chemical reaction network as a continuous time Markov chain (and its computational solution via the Gillespie algorithm (Gillespie 1977)) cannot typically be applied.

To proceed, we abstract the biological system to a chemical reaction network and use techniques from the mathematical modelling of chemical reaction networks. However, for biological interpretations it is imperative to remember that many cellular processes are governed by biochemical reactions (Alberts et al. 2007) and, hence, can be described by chemical reaction networks. First, we note that deterministic pattern formation reaction diffusion systems in biological applications with *n* interacting biochemical species take the form Turing (1952); Murray (2008)

1 $$\begin{aligned} \frac{\partial {\varvec{x}}}{\partial t} = {\varvec{D}}\frac{\partial ^2 {\varvec{x}}}{\partial s^2} + {\varvec{f}}({\varvec{x}}) \end{aligned}$$

in one spatial dimension, where ${\varvec{x}} \in \mathbb {R}^n_{\ge 0}$ is a vector of *n* chemical concentrations. The models we consider are networks with *mass action kinetics* (Horn and Jackson 1972), rendering the term ${\varvec{f}}({\varvec{x}}) = (f_1({\varvec{x}}),\ldots ,f_n({\varvec{x}}))^\mathrm{T}$ a vector of polynomial functions. The polynomials describe the underlying reaction network between the species, and ${\varvec{D}}(\partial ^2 {\varvec{x}}/\partial s^2)$ with ${\varvec{D}} = \text {diag}(D_1,\ldots ,D_n)$ describes the diffusion of ${\varvec{x}}$. Let there be *M* chemical reactions in the spatially homogeneous system whose dynamics are described by $\partial {\varvec{x}}/\partial t = {\varvec{f}}({\varvec{x}})$. Each reaction is parameterised by a rate constant $k_i > 0$, and therefore, we have a vector of rate constants ${\varvec{k}}=(k_1,\ldots ,k_M) \in \mathbb {R}^M_{> 0}$. Throughout this paper, we use homogeneous Neumann boundary conditions

2 $$\begin{aligned} \frac{\partial {\varvec{x}}}{\partial s}\biggr |_{s=0,L} = 0, \end{aligned}$$

where *L* is the domain length. Note that, although our work is only applied to systems of one spatial dimension, the theory can easily be extended to an arbitrary number of spatial dimensions. To do so, one replaces the one-dimensional diffusion term ${\varvec{D}} \partial ^2 {\varvec{x}}/\partial s^2$ with ${\varvec{D}}\nabla ^2{\varvec{x}}$, where $\nabla ^2$ is the *d*-dimensional Laplacian relative to the coordinates $s_i$ and $i\in \{1,\ldots ,d\}$.

Counter-intuitively, it has been shown that under certain conditions (Murray 2008), diffusion can drive an otherwise spatially uniform stable state to instability. Such unstable systems, which can form stable patterns, such as stripes or spots, are called *Turing systems* (Turing 1952; Woolley et al. 2017). To avoid the complications of bistable dynamics, and later stochastic analogues such as switching between steady states, we impose the constraint that the spatially homogeneous solution has a unique stable steady state, ${\varvec{x}}^*$, such that ${\varvec{f}}({\varvec{x}}^*) = 0$ (Murray 2008). To find models which have this property, we use techniques from real algebraic geometry (Müller et al. 2016) which provides simple tools, ensuring that there exists only a single steady state in a model. Since Turing’s work in 1952, many biological patterning systems have been suggested to be Turing systems (Castets et al. 1990; Cartwright 2002; Kondo and Miura 2010).

Fundamentally, the application of a set of partial differential equation (PDE) models for a biochemical reaction system assumes that the species of interest are in high enough concentration to allow continuum modelling. By contrast, when the number of particles in the biological system is low, intrinsic stochasticity of the system must be included in the model (Schumacher et al. 2013; Woolley et al. 2012; McKane and Newman 2005; McKane et al. 2014; Biancalani et al. 2011), which typically yields studies that investigate the impact of white noise.

However, as mentioned, stochasticity in biological systems can also arise from extrinsic noise and hence temporal variations in parameter values (Picco et al. 2017). Thus, instead we generalise the deterministic system to include *stochastic* parameter values. Specifically, we focus on the effect of stochastic fluctuations of reactions of the form $\emptyset \rightarrow X$, resulting in a constant term in the chemical reaction network ${\varvec{f}}({\varvec{x}})$, the “inflow” term. As the extrinsic noise can arise from a vast number of different mechanisms, such as varying experimental conditions (Lenive et al. 2016), it is largely free of microscopic constraints, especially the absence of temporal correlation. However, the impact of correlated external noise has, to the authors’ knowledge, received little theoretical modelling attention. Thus, we proceed to develop a framework to study external coloured noise forcing of the above deterministic system for pattern formation in biologically motivated scenarios, analysing how temporal correlation in noise, as described by colour, impacts self-organisation properties.

The paper is organised as follows. In Sect. 2, we introduce required notions of chemical reaction network theory to select a class of models relevant to our framework, i.e. those whose number of steady states is unaltered by changes in parameter values, temporal or otherwise. We then introduce stochastic inflow parameters and describe their connection coloured noise. In Sect. 3, we highlight the impact of noise colouring on the spatio-temporal patterns formed by example of the Schnakenberg system. In particular, we discuss noise arising from stochastic subnetworks and varying experimental conditions. Our numerical results are discussed in Sect. 4 where we summarise the distinguishable differences between the various noise colours.

## Theoretical Background and Power Spectra Analysis

In this section, we introduce the theoretical background of this paper.

### Chemical Reaction Network Theory

A central aim of chemical reaction network theory (CRNT) is to describe the properties of a chemical reaction network from its reaction graph alone (Feinberg 1987, 1988). One such property is the capacity for multiple steady states. Define a chemical reaction network by the multi-set ${\mathcal {N}} = \{{\mathcal {S}}, {\mathcal {C}}, {\mathcal {R}}\}$, where ${\mathcal {S}}, {\mathcal {C}}, {\mathcal {R}}$ are defined below. We begin by embedding the network into *n*-dimensional Euclidean space $\mathbb {R}^n$ by associating a basis vector ${\varvec{e}}_i$ to each chemical species $X_i$ such that $X_1\rightarrow {\varvec{e}}_1 = (1,0,0,\ldots )^\mathrm{T}$, $X_2\rightarrow {\varvec{e}}_2 = (0,1,0,\ldots )^\mathrm{T}$ and so on. Let ${\mathcal {S}} = \{X_1,\ldots ,X_n\}$ be the set of all chemical species in the network, then a generic reaction can be expressed as

3 $$\begin{aligned} \sum _{i=1}^n \alpha _i X_i \xrightarrow {k} \sum _{i=1}^n \beta _i X_i\; . \end{aligned}$$

The constants $\alpha _i$ and $\beta _i$ are *stoichiometric coefficients* which give information about how many molecules of $X_i$ are consumed and produced in each reaction. By letting $X_i\rightarrow {\varvec{e}}_i$, we can formulate a *reaction vector* describing the consumption, or production, of a species $X_i$ in a reaction

4 $$\begin{aligned} {\varvec{r}} = \sum _{i=1}^n (\beta _i-\alpha _i){\varvec{e}}_i. \end{aligned}$$

If an entry of ${\varvec{r}}$ is negative, then a species is consumed, whilst if an entry of ${\varvec{r}}$ is positive, then a species is produced.

Denote the set of all reaction vectors in a network by ${\mathcal {R}} = \{{\varvec{r}}_1,\ldots ,{\varvec{r}}_M\}$. To complete the description of a chemical reaction network in terms of sets and their embedding into Euclidean space, we introduce the notion of *complexes*. Complexes are linear combinations of species which appear on the left- or right-hand sides of reaction vectors. In Eq. (3), the two complexes are $C_1 = \sum _{i=1}^n \alpha _i X_i$ and $C_2 = \sum _{i=1}^n \beta _i X_i$, where $C_1$ is the *reactant complex* and $C_2$ is the *product complex*. Let the set of all complexes be ${\mathcal {C}} = \{C_1,\ldots ,C_l\}$. The reaction network, ${\mathcal {N}}$, is therefore a directed graph with vertex set ${\mathcal {C}}$ and a directed edge between vertices $C_i$ and $C_j$ if and only if $C_j-C_i \in {\mathcal {R}}$, with the same embedding, $X_i\rightarrow {\varvec{e}}_i$. Note that the description of the reaction network *does not* include any notion of rate constants *k*; however, many results in chemical reaction network theory include the rate constants explicitly as a positive vector ${\varvec{k}} = (k_1,\ldots ,k_m)^\mathrm{T}\in \mathbb {R}^M_{>0}$.

#### Example 1

(Schnakenberg) The (non-spatial) Schnakenberg system is one of the simplest chemical reaction networks whose dynamics exhibit a Hopf bifurcation and, therefore, present rich dynamical behaviour (Schnakenberg 1979). The system follows the reaction scheme5with species ${\mathcal {S}}= \{X_1,X_2\}$ and complexes ${\mathcal {C}} = \{\emptyset ,X_1,X_2,2X_1+X_2,3X_1\}$.

In this paper, we study the influence of noise on reaction–diffusion systems that are able to produce patterns in a well-defined parameter region. In particular, we study the Schnakenberg system. Critically, we will see that coloured noise is able to have both a constructive influence outside of this previously defined parameter region (i.e. the noise stabilises patterns where we would not expect them deterministically) and a destructive influence on patterns which normally would arise. In particular, we would like to exclude the intrinsically stochastic effect of *stochastic switching*, which occurs for a system that has multiple steady states in some parameter region. Stochastic switching describes the phenomenon that a chemical reaction network can jump from one steady state to another when subject to finite stochastic perturbations. To avoid this scenario, we use a network structure-based tool described in Müller et al. (2016) which a priori excludes multistationarity.

Take a chemical reaction network ${\mathcal {N}}$ and embed its complexes and reactions into ${\mathbb {R}}^n$. Then, define the matrices $m_1 = ({\varvec{r}}_1 {\varvec{r}}_2 \ldots {\varvec{r}}_M)$ for $r_i\in {\mathcal {R}}$ and $m_2^\mathrm{T} = ({\varvec{b}}_1 {\varvec{b}}_2\ldots {\varvec{b}}_r)$ where $\{{\varvec{b}}_i\}$ is the set of reactant complex vectors of ${\mathcal {N}}$. Let $\mathrm{diag}(k_1,\ldots ,k_M)$ be the diagonal matrix of reaction constants. Using the law of mass action, the dynamics of the species concentrations can be described by

6 $$\begin{aligned} \frac{\hbox {d}{\varvec{x}}}{\hbox {d}t} = {\varvec{f}}({\varvec{x}}) = m_1\,\mathrm{diag}(k_1,\ldots ,k_m)\, {\varvec{x}}^{m_2}, \end{aligned}$$

where ${\varvec{x}}^{m_2} = (x_1^{b_{11}}x_2^{b_{12}}\ldots x_n^{b_{1n}},\ldots ,x_1^{b_{M1}}x_2^{b_{M2}}\ldots x_n^{b_{Mn}})^\mathrm{T}$. Note that the reactant complexes and the reactions are structural properties of the reaction graph. Further, let ${\varvec{a}} \in {\mathbb {R}}^n$ and define its sign vector $\sigma ({\varvec{a}}) = \{-,0,+\}^n$ by applying the sign function to each component of ${\varvec{a}}$. Therefore, the *i*th component of the sign vector $\sigma ({\varvec{a}})_i = \text {sign}(a_i) \in \{+,0,-\}$.

The link from network structure to multistationarity is outlined in Müller et al. (2016, Theorem 1.4), and it concerns the injectivity of the map ${\varvec{f}}: {\varvec{x}}\mapsto d{\varvec{x}}/dt$. If ${\varvec{f}}({\varvec{x}})$ is injective, then there exists a unique vector ${\varvec{x}}^*\in {\mathbb {R}}^n$ such that ${\varvec{f}}({\varvec{x}}^*) = 0$. In other words, the network is monostationary. The conditions for an injective ${\varvec{f}}({\varvec{x}})$ hold for all parameter values ${\varvec{k}} = (k_1,\ldots ,k_M)$. A corollary of Müller et al. (2016, Theorem 1.4) is that ${\varvec{f}}({\varvec{x}})$ is injective if

7 $$\begin{aligned} \text {ker}(m_2) = \{0\} \text { and } \sigma (\text {ker}(m_1))\cap \sigma (\text {im}(m_2)) = \{0\}. \end{aligned}$$

Note that this condition depends on the network structure, specifically the spaces spanned by the kernel of the reaction matrix, $m_1$, and the image of the source complex matrix, $m_2$, but not on the parameter values.

#### Example 2

(Schnakenberg (cont.)) We check for a potential multistationarity in the Schnakenberg system by formulating the matrices $m_1$ and $m_2$.

8 $$\begin{aligned} m_1 = \begin{pmatrix} 1 &{} -1 &{} 0 &{} 1\\ 0 &{} 0 &{} 1 &{} -1 \end{pmatrix} \quad \quad \text {and}\quad \quad m_2 = \begin{pmatrix} 0 &{} 0\\ 1 &{} 0\\ 0 &{} 0\\ 2 &{} 1 \end{pmatrix}. \end{aligned}$$

It is easy to confirm that $\text {ker}(m_2) = \{0\}$. Further, we have

$$\begin{aligned}&\sigma (\text {ker}(m_1)) = \{(+,+,0,0)^\mathrm{T},(-,0,+,+)^\mathrm{T}\} \end{aligned}$$

and

9 $$\begin{aligned}&\sigma (\text {im}(m_2)) = \{(0,+,0,+)^\mathrm{T},(0,0,0,+)^\mathrm{T}\}. \end{aligned}$$

Therefore, the Schnakenberg system is monostationary for all choices of (positive) parameter values.

### Stochastic Inflow in Chemical Reaction Networks

We will now show how chemical reaction networks (CRNs) that satisfy injectivity (preclusion of multistationarity) can be applied to consider parameter stochasticity, especially to the case of stochastic inflows. In particular, this subsection serves to show the mathematical equivalence between stochastic inflows and internal noise systems but also to introduce a novel way of modelling external noise in chemical reaction networks. Readers interested in applying coloured noise to stochastic reaction–diffusion equations with internal noise modelled by reaction–diffusion PDEs or chemical Langevin equations may skip this subsection.

Much effort in the CRNT literature has been made to investigate “fully open systems”; these are CRNs in which every species has an inflow and an outflow reaction (Banerjee and Bhattacharyya 2014; Joshi and Shiu 2014; Félix et al. 2016; Banaji and Pantea 2018). By contrast, here we change the nature of the model by generalising the inflow rate constants to stochastic processes, rather than simply adding inflow processes as deterministic reactions. It should be noted that despite the name we do not assume that these reactions are inflows into the domain of simulation; rather, the inflow reactions correspond to the creation of particles from some other process which happens *throughout the domain*. Sometimes, these inflow reactions are called “inputs” in engineering applications and the concentrations are the “outputs”, and therefore, the study of chemical reaction networks with varying inflows can be rephrased into a study of the “input–output” relation as has been the approach in previous work (Frank 2013).

Consider a chemical reaction network ${\mathcal {N}}$. An *inflow* reaction for species $X_i$ is manifested in the reaction graph as

$$\begin{aligned} \emptyset \xrightarrow {k_{\text {in}}} X_i\;. \end{aligned}$$

**Fig. 1:**
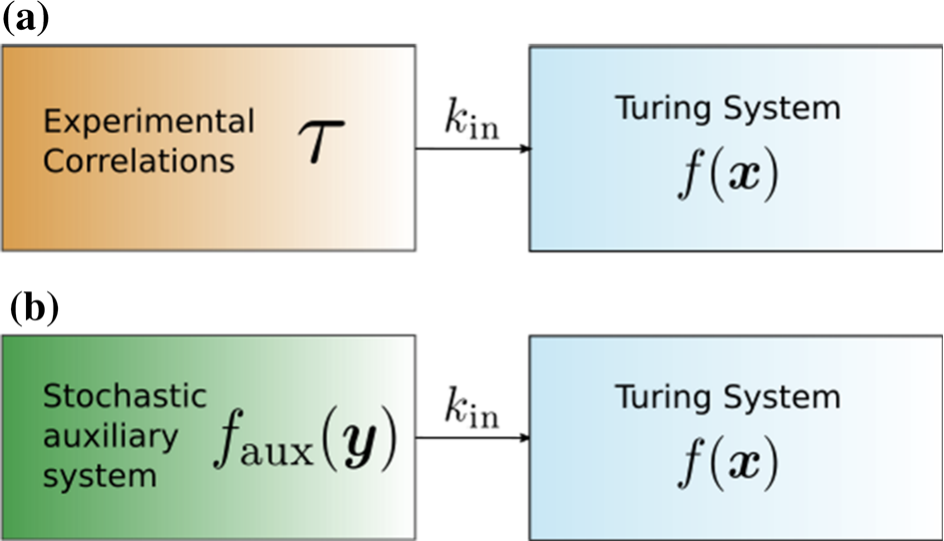
The two mechanisms contributing to stochastic inflows. The boxes on the left are usually treated as black boxes, resulting in some constant inflow modelled by the parameter $k_\text {in}$. We differentiate between experimental fluctuations, symbolised by a correlation $\tau$ in (**a**) and auxiliary networks described by $f_\text {aux}({\varvec{y}})$ in case (**b**) (Color figure online)

Usually, the work in CRNT assumes the rate constant $k_{\text {in}}$ to be constant in time (and space). However, the zero complex, $\emptyset$, symbolises a process not included in the model such as the production of $X_i$ by another network, which we call an “auxiliary network”, or a mechanical addition of $X_i$ to an experimental set-up. Both mechanisms are often subsumed into $\emptyset$ and usually approximated as constant or “perfect” inflow; however, they can exhibit intricate dynamics. We model the dynamics of “non-perfect” inflows by a stochastic process whose origin can be twofold (Fig. 1):

- *Experimental fluctuations*: In chemical engineering, it is assumed that inflow of chemicals into a reactor is a perfectly deterministic process; however, due to mechanical, or other experimental imperfections, the inflow into a reaction can vary stochastically. This, again, renders the influx parameter $k_{\text {in}}$ into a stochastic process $K_\text {in}(t)$.
- *Stochastic subsystems*: We assume that the inflow into the deterministic reaction diffusion system is proportional to the concentration of a chemical in another chemical reaction network with a unique fixed point, the *auxiliary network*. When the number of particles in the auxiliary network is large, the auxiliary system is at steady state and the inflow rate $k_\text {in}$ is constant. However, when the particle number in the auxiliary system is low, stochastic fluctuations cannot be ignored and, whilst still proportional to the concentration of a species in the auxiliary system, the actual influx parameter $k_\text {in}$ is a stochastic process $K_\text {in}(t)$. Due to correlations and interactions within the auxiliary system, $K_\text {in}(t)$ may not simply be white noise. In this paper, we assume that the particle number of the auxiliary network is in the *weak noise* regime (van Kampen 1983).

Whereas the two sources of parameter noise are conceptually different, their mathematical description is the same. Consider a stochastic process *K*(*t*) with an underlying distribution ${\mathcal {D}}(t)$. To be biologically relevant (i.e. to ensure all chemical concentrations are non-negative at every *t*), we require ${\mathcal {D}} = 0$ for all $K(t) < 0$ and, hence, the distribution ${\mathcal {D}}$ needs to be continuous almost everywhere. To simplify the following mathematical analysis, we approximate the distribution of *K*(*t*) as a *truncated Gaussian*, since we would like to connect our analysis to the internal noise case. Future work could focus on other non-negative distributions, such as the log-normal distribution, on a continuous probability space. Therefore, we can describe *K*(*t*) by

$$\begin{aligned} K(t)&= k + \frac{1}{\sqrt{\varOmega }} \eta (t), \end{aligned}$$

with

10 $$\begin{aligned} \langle \eta (t)\rangle \approx 0\quad&\text {and}\quad \langle \eta (t),\eta (t')\rangle = g(t,t'), \end{aligned}$$

where $g(t,t')$ is a positive function and $\eta$ follows a (truncated) Gaussian distribution whose non-truncated mean is exactly zero. We assume that the truncation is small such that in analytic calculations the truncated moments can be approximated by the full moments of $\eta$. Due to the negligible truncation, we assume that the moments of the truncated Gaussian can be approximated by the moments of the full Gaussian, such that $\langle K(t)-k\rangle = 0$ and $\langle (K(t)-k)(K(t')-k)\rangle = \varOmega ^{-1} g(t,t')$. In other words, the small exponential tail which is truncated would only provide a negligible perturbation to the moments of the non-truncated Gaussian. The validity of this assumption is confirmed in the computational results of Sect. 3. In the remainder of this paper, we will show how various functions $g(t,t')$ can arise in mathematical modelling, especially when stochastic auxiliary networks are considered.

We can further connect the parameter $\varOmega$ to physical quantities of the underlying chemical reaction network by again considering the sources of stochastic inflow. We assume that the origin of the stochastic inflow is stochastic auxiliary networks or experimental imperfections. In the limit of either a perfect experiment or a deterministic auxiliary network, the inflow should be deterministic and $\langle (K(t)-k)(K(t')-k)\rangle = 0$ implying $\varOmega \rightarrow \infty$. The parameter $\varOmega$, therefore, represents the noise strength. Large $\varOmega$ (small noise) is required for the assumptions on the moments of the truncated Gaussians to hold.

Next, consider a chemical reaction network ${\mathcal {N}}$ in which a subset of species has an inflow reaction. Denote the set of species with (stochastic) inflow by ${\mathcal {S}}_\text {in} \subseteq {\mathcal {S}}$. If there is more than one (stochastic) inflow $|{\mathcal {S}}_\text {in}|>1$, the stochastic process will be a multi-dimensional version of (10). Further, assume that the stochastic process has a spatial dependence. The description of the stochastic process outlined in this section still applies in this case, if we have ${\varvec{K}}_\text {in}(s,t)$ as the vector of stochastic inflow such that

$$\begin{aligned} {\varvec{K}}_\text {in}(s,t)&= {\varvec{k}}_{\text {in}} + \frac{1}{\sqrt{\varOmega }}{\varvec{\eta }}(s,t), \end{aligned}$$

with

11 $$\begin{aligned} \langle {\varvec{\eta }}(s,t)\rangle&\approx 0\nonumber \\ \langle {\varvec{\eta }}(s,t){\varvec{\eta }}^\mathrm{T}(s',t')\rangle&= B(s,s')G(t,t')\nonumber \\&= \{B_{ij}(s,s')G_{ij}(t,t')\}, \end{aligned}$$

where $B(s,s')$ represents the potential covariances between the stochastic inflows and $G(t,t')$ models the temporal correlations.

Since our assumptions made the inflow process additive and uncoupled to the species, the stochastic reaction network can be described by the system of stochastic partial differential equations (SPDEs)

12 $$\begin{aligned} \frac{\partial {\varvec{x}}}{\partial t} = D\frac{\partial ^2 {\varvec{x}}}{\partial s^2} + {\varvec{f}}({\varvec{x}}) + \frac{1}{\sqrt{\varOmega }}{\varvec{\eta }}, \end{aligned}$$

where ${\varvec{\eta }}$ is a vector of stochastic processes such that its support is $supp({\varvec{\eta }}) = {\mathcal {S}}_\text {in}$.

Note that in our limit randomising inflows leaves ${\mathcal {N}}$ as well as ${\varvec{f}}({\varvec{x}})$ invariant, and hence, the steady-state structure of the system; i.e. if we start out with an injective function ${\varvec{f}}({\varvec{x}})$, then adding stochasticity will not change this injectivity.

The assumption of rate constants being stochastic processes rather than constants can be extended to non-inflow reactions too. However, Eq. (12) will have extra terms of multiplicative noise (see Example 3). In the next section, we study perturbations to the steady state whose scaling will render the study of non-inflow stochastic rate constants a trivial extension of the analysis of this subsection.

#### Example 3

(Non-inflow noise) Suppose that in the Schnakenberg model the non-inflow reaction

$$\begin{aligned} 2X_1 + X_2 \xrightarrow {k_3} 3X_1 \end{aligned}$$

is subject to external noise. Hence, the rate constant $k_3$ becomes a stochastic process $k_3\rightarrow K_3$, and applying similar modelling assumptions as for inflow reactions, we assume that $K_3$ follows a truncated Gaussian distribution whose moments can be approximated by the moments of the full Gaussian. Hence, we let $K_3\approx k_3 + 1/\sqrt{\varOmega }\; \eta$ as before. Substituting $k_3\rightarrow k_3 + 1/\sqrt{\varOmega }\; \eta$ into the governing equations for the Schnakenberg system yields a model with multiplicative external noise.

Further, it should be added that in this paper it is assumed that in the reaction–diffusion system internal noise due to low particle numbers is negligible compared to the effects of external noise which arises from the rate constants being stochastic processes. However, if internal noise was to be included, then the reaction–diffusion system would have to be described by a chemical master equation with stochastic inflow rate constants.

### Power Spectra for Stochastic Inflows

To fully classify the patterns arising from the addition of stochastic inflows, we calculate the power spectra of the patterns. Power spectra are an analytic tool showing which spatial and temporal frequencies are present in a pattern (Adamer et al. 2017; Schumacher et al. 2013; Woolley et al. 2011b). Peaks in power spectra give information about dominant frequencies and, hence, about oscillatory behaviour of a system in space and time.

First, we linearise equation (12) about the fixed point ${\varvec{x}} \sim {\varvec{x}}^* + \overline{\delta {\varvec{x}}}$, where $\overline{\delta {\varvec{x}}}$ represent small perturbations. These perturbations should decay in the deterministic limit as the system will converge to the steady state outside the Turing parameter regime. Inside the Turing regime, the system is assumed to converge to a stable pattern rather than a stable state which is not considered in this paper. Hence, our analysis is valid only *outside* the Turing pattern regime. Substituting the linearisation ansatz into Eq. (12) and keeping lowest order terms only, we get

13 $$\begin{aligned} \frac{\partial \overline{\delta {\varvec{x}}}}{\partial t} = D\frac{\partial ^2 \overline{\delta {\varvec{x}}}}{\partial s^2} + J|_{{\varvec{x}}={\varvec{x}}^*}\overline{\delta {\varvec{x}}} + \frac{1}{\sqrt{\varOmega }}{\varvec{\eta }}, \end{aligned}$$

where $J|_{{\varvec{x}}={\varvec{x}}^*}$ is the Jacobian of ${\varvec{f}}({\varvec{x}})$ evaluated at the fixed point ${\varvec{x}}^*$, which for notational convenience we will denote as *J*.

We make one further assumption, namely the scaling of the perturbation $\overline{\delta {\varvec{x}}}$ with the parameter $\varOmega$ controlling the magnitude of the stochastic input. Let $\overline{\delta {\varvec{x}}} = \varOmega ^\alpha \delta {\varvec{x}}$. This gives

14 $$\begin{aligned} \varOmega ^\alpha \frac{\partial \delta {\varvec{x}}}{\partial t} = \varOmega ^\alpha D\frac{\partial ^2 \delta {\varvec{x}}}{\partial s^2} + \varOmega ^\alpha J\delta {\varvec{x}} + \varOmega ^{-\tfrac{1}{2}}{\varvec{\eta }}. \end{aligned}$$

Then, if $\alpha < -1/2$ and in the limit $\varOmega \rightarrow \infty$, the leading-order term is the stochastic process ${\varvec{\eta }}$ only. Equally, if $\alpha > -1/2$, then ${\varvec{\eta }}$ could be neglected to leading order in $\varOmega$. Hence, we let $\alpha = -1/2$ for a dominant balance and Eq. (14) simplifies to

15 $$\begin{aligned} \frac{\partial \delta {\varvec{x}}}{\partial t} = D\frac{\partial ^2 \delta {\varvec{x}}}{\partial s^2} + J\delta {\varvec{x}} + {\varvec{\eta }}. \end{aligned}$$

The reasoning behind choosing $\alpha = -1/2$ is analogous to the reasoning of the scaling in the weak noise expansion of van Kampen (1983), especially the requirement that in the limit $\varOmega \rightarrow \infty$ the noise correlations are zero. However, the system size in the external noise case is imposed by an external mechanism such as a non-modelled stochastic network with system size $\varOmega$.

The scaling of the perturbations to the steady state determines a hierarchy on the terms of Eq. (12), and in the case of non-inflow stochastic rate constants, all multiplicative noise terms are linearised to additive terms with all non-trivial multiplicative perturbations only manifesting at higher order. The approach of this paper is similar to a weak noise approximation, however, with the fundamental difference that the weak noise expansion is used to approximate a probability distribution, whereas the perturbations to the steady state used in this paper approximate the local dynamics of a systems of PDEs.

Note that the parameter $\varOmega$ controls the amplitude of the perturbation to the steady state and, therefore, is also responsible for size of the amplitude of any patterns one might observe. Increasing $\varOmega$ decreases the amplitude of the patterns. If $\varOmega$ is too small, then the perturbations ${\varvec{\overline{\delta x}}}$ are not small and the linear approximation breaks down, i.e. the concentrations of the chemicals go negative.

Note that Eq. (15) is mathematically equivalent to a chemical Langevin equation of compartmentalised diffusion in the limit of the compartment size, $\varDelta _s$, going to zero (Smith 1985). However, in our derivation the source of the noise is *external*. To emphasise the mathematical connection with internal noise, we will in fact discretise equation (15) into a finite number of compartments, such that for an *n* species network with ${\mathcal {K}}$ compartments we have

16a $$\begin{aligned} \delta {\varvec{x}}&= [\delta x_1,\ldots ,\delta x_{\mathcal {K}},\delta x_{{\mathcal {K}}+1},\ldots , \delta x_{2{\mathcal {K}}},\ldots ,\delta x_{n{\mathcal {K}}}]^\mathrm{T}, \end{aligned}$$

16b $$\begin{aligned} {\varvec{\eta }}&= [\eta _1,\ldots ,\eta _{\mathcal {K}},\eta _{{\mathcal {K}}+1},\ldots , \eta _{2{\mathcal {K}}},\ldots ,\eta _{n{\mathcal {K}}}]^\mathrm{T},\end{aligned}$$

16c $$\begin{aligned} s&\approx s_i = i\varDelta _s\;\;\;\; \text {where } i = \{1,\ldots ,{\mathcal {K}}\},\end{aligned}$$

16d $$\begin{aligned} \frac{\partial ^2 \delta {\varvec{x}}(s)}{\partial s^2}&\approx \frac{\delta {\varvec{x}}(s_i+\varDelta _s) + \delta {\varvec{x}}(s_i-\varDelta _s) - 2\delta {\varvec{x}}(s_i)}{(\varDelta _s)^2}, \end{aligned}$$

and the matrices *D* and *J* turn into block matrices such that

17 $$\begin{aligned} D = \begin{pmatrix} \left[ D_1\right] _{{\mathcal {K}}\times {\mathcal {K}}} &{} 0 &{} 0 &{} \cdots \\ 0 &{} \left[ D_2\right] _{{\mathcal {K}}\times {\mathcal {K}}} &{} 0 &{} \cdots \\ \vdots &{} \vdots &{} \vdots &{} \ddots \end{pmatrix}. \end{aligned}$$

The $ij^{\text {th}}$ block of *J* is $[J_{ij}]_{{\mathcal {K}}\times {\mathcal {K}}}$ to give

18 $$\begin{aligned} J = \begin{pmatrix} \left[ J_{11}\right] _{{\mathcal {K}}\times {\mathcal {K}}} &{} \left[ J_{12}\right] _{{\mathcal {K}}\times {\mathcal {K}}} &{} \cdots \\ \left[ J_{21}\right] _{{\mathcal {K}}\times {\mathcal {K}}} &{} \left[ J_{22}\right] _{{\mathcal {K}}\times {\mathcal {K}}} &{} \cdots \\ \left[ J_{31}\right] _{{\mathcal {K}}\times {\mathcal {K}}} &{} \left[ J_{32}\right] _{{\mathcal {K}}\times {\mathcal {K}}} &{} \cdots \\ \vdots &{} \vdots &{} \ddots \end{pmatrix}. \end{aligned}$$

Compartmentalisation results in a system of $n{\mathcal {K}}$ coupled SDEs

19 $$\begin{aligned} d\delta {\varvec{x}} = A\delta {\varvec{x}}\,dt + {\varvec{\eta }}\, dt, \end{aligned}$$

with $A= D/\varDelta _s^2 + J$, $\langle \eta _i(t)\rangle = 0$ and $\langle \eta _i(t)\eta _j(t')\rangle = B_{ij}G_{ij}(t,t')$. Note that after compartmentalisation $B(s,s')$ is a block matrix of $n^2$ ${\mathcal {K}}\times {\mathcal {K}}$ matrices, describing the spatial auto-correlation of each species and the spatial correlations between species.

To calculate the power spectra of the system, we introduce the discrete cosine transform (Briggs and Henson 1995)

20 $$\begin{aligned} f_\kappa = \varDelta _s\sum _{j=1}^{\mathcal {K}} \cos [\kappa \varDelta _s(j-1)]f(s_j). \end{aligned}$$

The cosine transform incorporates the Neumann (no flux) boundary conditions, which are commonly used for reaction–diffusion systems; however, other boundary conditions can easily be implemented (Briggs and Henson 1995). Due to the boundary conditions, we require $\kappa = m\pi /l$ with $m \in \{0,1,2,\ldots \}$. We refer to *m* as the *spatial mode*. Note that the use of $(j-1)$ instead of *j* which is due to the fact that the compartment labelling starts at $j=1$. Hence, by applying the spatial Fourier transform we reduce the system of $n{\mathcal {K}}$ SDEs (19) to a system of *n* coupled SDEs

21 $$\begin{aligned} \hbox {d}\delta {\varvec{x}}_\kappa = A_\kappa \delta {\varvec{x}}_\kappa \, \hbox {d}t + {\varvec{\eta }}_\kappa \, \hbox {d}t. \end{aligned}$$

Finally, applying the temporal Fourier transform

22 $$\begin{aligned} {\tilde{f}}(\omega ) = \int _{-\infty }^\infty f(t)e^{-i\omega t}\hbox {d}t, \end{aligned}$$

we get an expression for $\delta \tilde{{\varvec{x}}}_\kappa (\omega )$. Note that the Fourier transform always exists if we consider the system to have a fixed point (Woolley et al. 2011c).

23 $$\begin{aligned} \delta \tilde{{\varvec{x}}}_\kappa (\omega ) = \left[ -A_\kappa -i\omega \right] ^{-1}\tilde{{\varvec{\eta }}}_\kappa (\omega ). \end{aligned}$$

Therefore, the power spectra of the pattern, $P_{\delta x}(\kappa ,\omega )$, can be expressed as the diagonal elements of

24 $$\begin{aligned} \langle \delta \tilde{{\varvec{x}}}_\kappa (\omega )\delta \tilde{{\varvec{x}}}_\kappa ^\dagger (\omega )\rangle = \varPhi ^{-1}\underbrace{\langle \tilde{{\varvec{\eta }}}_\kappa (\omega )\tilde{{\varvec{\eta }}}_\kappa ^\dagger (\omega )\rangle }_{N} \left( \varPhi ^{-1}\right) ^\dagger , \end{aligned}$$

where we defined

25 $$\begin{aligned} \varPhi = -\left[ A_\kappa + i\omega \right] , \end{aligned}$$

and $\dagger$ denotes the Hermitian conjugate of a matrix. In order to be guaranteed real power spectra, we need $N = N^\dagger$. In the case of Gaussian noise, we have

26 $$\begin{aligned} N = {\mathcal {F}}_\text {cos}\left( {\mathcal {F}}(BG(t,t'))\right) , \end{aligned}$$

where ${\mathcal {F}}_\text {cos}$ denotes the cosine transform and ${\mathcal {F}}$ the temporal Fourier transform. Note that when all temporal correlations are equal such that $G_{ij}(t,t') = g(t,t')$ the transform factor becomes

27 $$\begin{aligned} N = {\mathcal {F}}_\text {cos}\left( B\right) {\mathcal {F}}(g(t,t')) = B_\kappa P_\text {Correlations}(\omega ), \end{aligned}$$

where $P_{\text {Correlations}}$ is the power spectrum of the correlation function $g(t,t')$ and $B_\kappa$ is the $n\times n$ matrix of covariances. For white noise, we take ${\varvec{\eta }}\hbox {d}t = {\varvec{\xi }}\sqrt{\hbox {d}t}$ to reduce (19) to the Itô form

28 $$\begin{aligned} \hbox {d}\delta {\varvec{x}} = A\delta {\varvec{x}}\,\hbox {d}t + {\varvec{\xi }}\sqrt{\hbox {d}t}, \end{aligned}$$

with $\langle {\varvec{\xi }}\rangle = 0$ and $\langle \xi _i(t)\xi _j(t')\rangle = B_{ij}\delta (t-t')$ and, therefore, $P_{\text {Correlations}} = 1$. Hence, we see that for white noise the power spectra are

29 $$\begin{aligned} \langle \delta \tilde{{\varvec{x}}}_\kappa (\omega )\delta \tilde{{\varvec{x}}}_\kappa ^\dagger (\omega )\rangle&= \left[ \varPhi ^{-1}\langle \tilde{{\varvec{\eta }}}_\kappa (\omega )\tilde{{\varvec{\eta }}}_\kappa ^\dagger (\omega )\rangle \left( \varPhi ^{-1}\right) ^\dagger \right] \nonumber \\&= \left[ \varPhi ^{-1} B_\kappa \left( \varPhi ^{-1}\right) ^\dagger \right] = P_\text {white}. \end{aligned}$$

For general temporal correlations $g(t,t')$, we have

30 $$\begin{aligned} \langle \delta \tilde{{\varvec{x}}}_\kappa (\omega )\delta \tilde{{\varvec{x}}}_\kappa ^\dagger (\omega )\rangle&= \left[ \varPhi ^{-1} B_\kappa P_\text {Correlations}\left( \varPhi ^{-1}\right) ^\dagger \right] \nonumber \\&= P_\text {white}P_\text {Correlations}, \end{aligned}$$

such that the spectra of the noise colour appear as a multiplicative factor modulating the white noise spectra.

### Application to the Schnakenberg System

We illustrate our analysis via the example of the one-dimensional spatial Schnakenberg kinetics. The non-spatial model was discussed in Example 1. The Schnakenberg system is an $n=2$ species reaction–diffusion system which, despite its apparent simplicity, exhibits a wealth of different behaviours in the deterministic (Iron et al. 2004; Maini et al. 2012; Ward and Wei 2002; Flach et al. 2007) as well as stochastic (Schumacher et al. 2013; Woolley et al. 2011b) regimes. The Schnakenberg system is a system in which every species has an inflow reaction and, hence, both species can be subject to stochastic inflow. Hence, the Schnakenberg system is a perfect candidate to highlight the effects of coloured noise computationally and illustrate how the above analysis can be applied in practise.

The dynamics of the Schnakenberg system is described by the system of PDEs

31 $$\begin{aligned} \frac{\partial x_1}{\partial t}&= D_1\frac{\partial ^2 x_1}{\partial s^2} +k_1 - k_{-1} x_1 + k_3x_1^2x_2,\nonumber \\ \frac{\partial x_2}{\partial t}&= D_2\frac{\partial ^2 x_2}{\partial s^2} + k_2 - k_3x_1^2x_2. \end{aligned}$$

From Example 2, we see that the Schnakenberg system is monostationary for all positive parameter values and, therefore, stochastic fluctuations in the inflow parameters cannot trigger stochastic switching.

Next, we consider stochastic inflows, which result in $k_i \rightarrow k_i + 1/\sqrt{\varOmega }\eta _i$ for $i \in \{1,2\}$ and the equations

32 $$\begin{aligned} \frac{\partial x_1}{\partial t}&= D_1\frac{\partial ^2 x_1}{\partial s^2} +k_1 - k_{-1} x_1 + k_3x_1^2x_2 + \frac{1}{\sqrt{\varOmega }}\eta _1,\nonumber \\ \frac{\partial x_2}{\partial t}&= D_2\frac{\partial ^2 x_2}{\partial s^2} + k_2 - k_3x_1^2x_2 + \frac{1}{\sqrt{\varOmega }}\eta _2. \end{aligned}$$

Linearising equations (32) about the steady state $x^*_1 = (k_1 + k_2)k^{-1}_{-1}$, $x^*_2 = k_2k_{-1}^2k_3^{-1}(k_1+k_2)^{-2}$ and discretising space, we get a system of linear stochastic differential equations similar to the ones arising from the study of internal noise (Schumacher et al. 2013)

33 $$\begin{aligned} \frac{\hbox {d}{\varvec{x}}}{\hbox {d}t}&= A{\varvec{x}} + {\varvec{\eta }}, \end{aligned}$$

with

34 $$\begin{aligned} A&= \begin{pmatrix} {\varvec{a}} &{} {\varvec{b}}\\ {\varvec{c}} &{} {\varvec{d}} \end{pmatrix}, \end{aligned}$$

where ${\varvec{a}},{\varvec{d}}$ are tridiagonal matrices with diagonal elements

35 $$\begin{aligned} a_0&= -2D_1/\varDelta _s^2 -k_{-1}+2k_3x_1^*x_2^*,\nonumber \\ d_0&= - 2D_2/\varDelta _s^2 - k_3x_1^{*2}, \end{aligned}$$

and off-diagonal (sub- and super-diagonal) elements

36 $$\begin{aligned} a_1&= D_1/\varDelta _s^2 \equiv d_u,\nonumber \\ d_1&= D_2/\varDelta _s^2 \equiv d_v. \end{aligned}$$

The matrices ${\varvec{b}}$ and ${\varvec{c}}$ are diagonal matrices with entries

37 $$\begin{aligned} b_0&= k_3x_1^{*2},\nonumber \\ c_0&= -2k_3x_1^*x_2^*. \end{aligned}$$

Note that the vector ${\varvec{x}}$ has $2{\mathcal {K}}$ components. There are ${\mathcal {K}}$ components for species $x_1$ and ${\mathcal {K}}$ components for species $x_2$.

Hence, Fourier transforming Eq. (33) in space and time we can compute the power spectra,

38 $$\begin{aligned} P(\kappa ,\omega )&= \left[ A_\kappa +i\omega \right] ^{-1}\langle {\tilde{\eta }}_\kappa (\omega ){\tilde{\eta }}_\kappa ^\dagger (\omega )\rangle \left( \left[ A_\kappa +i\omega \right] ^{-1}\right) ^\dagger , \end{aligned}$$

with

39 $$\begin{aligned} A_\kappa&= \begin{pmatrix} a_0 + 2a_1\cos {\kappa \varDelta _s} &{} b_0\\ c_0 &{} d_0+2d_1\cos {\kappa \varDelta _s} \end{pmatrix}. \end{aligned}$$

In the following section, we will discuss the effect of various noise correlations on the power spectra, and hence, the patterns generated.

## Computational Results

In this section, we highlight the computational patterns generated by a Turing system with coloured noise (Table 1).

**Table 1:** An overview of our main results on various noise colours

Colour	Correlation function	Effect	Possible origin	Section
White	1	N/A	Internal	3.2
Ornstein–Uhlenbeck	$1/\left( \omega ^2\tau ^2+1\right)$	Suppresses small $\omega$	Experimental apparatus	3.3
Red	$1/\omega ^2$	Excites small $\omega$	Deterministic	3.4
Predator–prey	$\left( \alpha +\beta \omega ^2\right) /\left( \omega ^4 + \gamma \omega ^2+\delta \right)$	Induces oscillations	Stochastic subsystems	3.5

### Numerical Methods

The system of SDEs (19) is simulated by an Euler–Maruyama scheme (Peter 1995) with time step $\Delta t$ such that

40 $$\begin{aligned} {\varvec{x}}(t+\Delta t) = {\varvec{x}}(t) + {\varvec{Ax}}\Delta t + {\varvec{\eta }}\Delta t. \end{aligned}$$

The important step of the integration is to find the correct vector ${\varvec{\eta }}$.

The white noise and Ornstein–Uhlenbeck noise are generated by an auxiliary noise process. In particular, at teach time step the white noise is sampled from a multivariate Gaussian distribution

$$\begin{aligned} {\varvec{\eta }}\Delta t \sim {\mathcal {N}}(0, {\varvec{B}}\Delta t). \end{aligned}$$

The Ornstein–Uhlenbeck process is a generated by the stochastic differential equation

$$\begin{aligned} \frac{\hbox {d}{\varvec{\eta }}}{\hbox {d}t} = -\frac{1}{\tau }{\varvec{\eta }} + \frac{\sqrt{{\varvec{B}}}}{\tau }{\varvec{\xi }}, \end{aligned}$$

where ${\varvec{\xi }}$ is a standard white noise vector. Therefore, at time *t* the vector ${\varvec{\eta }}(t)$ is added to the system of SDEs. The Ornstein–Uhlenbeck “auxiliary equation” is integrated by an Euler–Maruyama scheme as described in Milshtein and Tret ’yakov (1994).

To simulate power law noise for which, in general, no auxiliary SDE exists, we use inverse transforms (Timmer and Koenig 1995). To generate a vector ${\varvec{\eta }}(t)$ with correlations $\langle {\varvec{\eta }}(t){\varvec{\eta }}^\mathrm{T}(t')\rangle = {\varvec{B}}g(t-t')$, we first use the fact that ${\varvec{\eta }} = \sqrt{{\varvec{B}}}{\varvec{\xi }}$ where ${\varvec{\xi }}$ may be correlated in time but not in space, i.e. $\langle {\varvec{\xi }}(t){\varvec{\xi }}^\mathrm{T}(t')\rangle = \delta _{ij} g(t-t')$. Then, let the power spectrum of $\xi _i$ be $1/\omega ^\alpha$ as described in Sect. 3.4 and use the algorithm of Timmer and Koenig (1995) to create a time series. Multiplication of ${\varvec{\xi }}(t)$ with $\sqrt{{\varvec{B}}}$ gives the desired noise process, ${\varvec{\eta }}(t)$, which can be added to the SDE system. Auxiliary networks as in 3.5 can be simulated by either method, and in this paper, the auxiliary network input is generated by using the inverse transforms technique.

The truncation of the Gaussian distribution is implemented by setting negative values of the noise to zero at each time step. However, negative inflows were a rare occasion (for the simulation parameters chosen, only for violet noise any truncation was needed) and, hence, truncation had no effect on our simulations.

### White Noise

First, we consider external white noise. In this special case, the noise vector ${\varvec{\eta }}$ is just a Wiener process with correlation matrix *B*. This case is mathematically identical to the case of internal noise in the weak noise limit as studied in Schumacher et al. (2013). The main differences between internal noise and the parameter noise considered in this paper are the amplitude of the noise and the exact forms of the covariances of the stochastic processes ${\varvec{\eta }}$.

Both approximations (the weak noise limit, as well as our “truncated Gaussian” approximation) assume that the stochastic effect is a perturbation to the deterministic limit. However, as derived in Schumacher et al. (2013), the covariance matrix of the stochastic processes ${\varvec{\eta }}$ is determined by the microscopic processes, whereas for external noise, whose origin can be manifold, the covariance matrix is arbitrary. For mathematical simplicity, we choose the covariance matrix

41 $$\begin{aligned} B_\kappa = \begin{pmatrix} 1 &{} 1\\ 1 &{} 1 \end{pmatrix}. \end{aligned}$$

Throughout the remainder of this paper, we fix the parameter values to

$$\begin{aligned} k_1&= k_2 = 10.0,\quad k_3 = 0.01, \quad k_{-1} = 20.0,\\ L&= 0.1,\quad {\mathcal {K}} = 40, \end{aligned}$$

with diffusion coefficients

42 $$\begin{aligned} D_1&= 10^{-4},\nonumber \\ D_2&= 10^{-2}, \end{aligned}$$

which results in steady-state concentrations of $x_1^* = 1$, $x_2^* = 1000$. The parameters chosen are not generic, but they represent a particularly interesting point in parameter space. The parameters are in the (stable) oscillatory regime of the Schnakenberg system and outside the Turing space. The main function of the noise will be to temporarily move the system into the Turing regime. Note that the system has large inflows (and outflows) compared to the chemical reaction parameterised by $k_3$ which allows for larger variations in the noise translating into larger pattern amplitudes. The modifications to the power spectra due to coloured noise are generic and apply all points in parameter space. However, the visibility of these modifications depends on the point in parameter space and the correlation matrix. Similarly, the results on amplitude of the patterns are parameter dependent. To simulate the system, we discretise the space into 40 compartments of width $\varDelta _s = 0.0025$ (which gives a total domain length of $L = 0.1$).

**Fig. 2:**
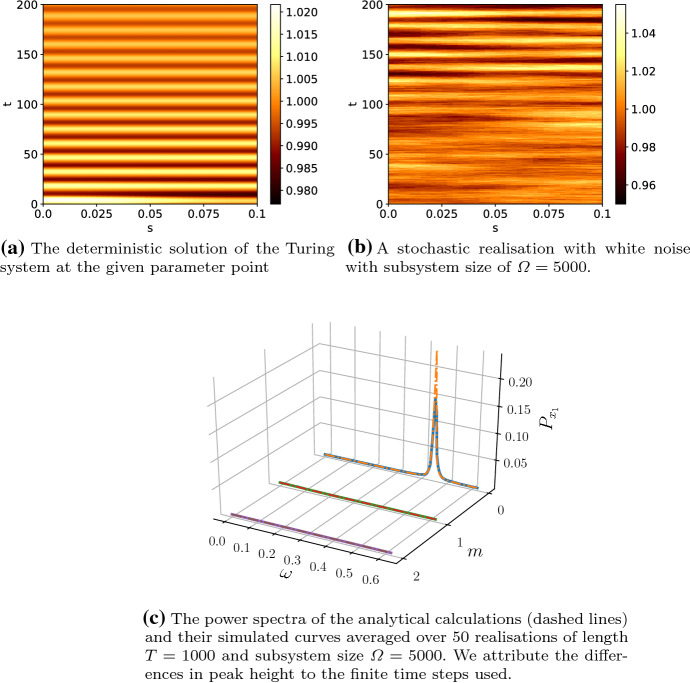
An overview of the deterministic and white noise behaviour of the system with parameters as in (42). The peak in 2c at zero spatial mode, $m=0$, indicates temporal oscillations, which result from deterministic limit cycle oscillations. Due to the choice of reaction kinetics, the temporal oscillations of species $x_1$ and $x_2$ are in phase (Color figure online)

Simulating Eq. (33) under the influence of white noise and with the given parameter values, we obtain Fig. 2 and its corresponding averaged power spectrum. The power spectrum shows the temporal frequencies, $\omega$, and spatial modes, *m*, present in the pattern. The amplitude of the oscillations is about 3% of the steady-state value for species $x_1$, whereas for species $x_2$ it is much lower. Therefore, we assume that any patterning of $x_2$ is not generally measurable and, therefore, we focus our attention on $x_1$. The prominent temporal oscillations with no spatial dependency manifest themselves as a sharp peak in the power spectrum. This is due to the fact that the chosen parameter point is in the oscillatory regime of the Schnakenberg model. Upon close inspection, slight spatial variations in the pattern of $x_1$ can be seen, but with white noise these are not very pronounced. In the following sections, we show how noise colour can enhance spatial modes, create additional oscillations or even create a stable pattern.

### Ornstein–Uhlenbeck Noise

Next, we investigate the effect of exponentially correlated noise also known as Ornstein–Uhlenbeck noise (Hanggi and Jung 1995). This stochastic process has a finite correlation time $\tau$ which we interpret as a response time. Consider an experiment in which the inflow rate is highly sensitive to the ambient temperature. Further, suppose this temperature undergoes random fluctuations about a regulated mean value. Therefore, the correlation time $\tau$ could represent the average response time of the temperature regulator.

We make the simplification that all the temporal correlations in Eq. (11) originate from Ornstein–Uhlenbeck processes with the same correlation time, $\tau$, such that

43 $$\begin{aligned} g_{ij}(t,t') = g(t,t') = \frac{1}{2\tau }e^{-\tfrac{|t-t'|}{\tau }}. \end{aligned}$$

**Fig. 3:**
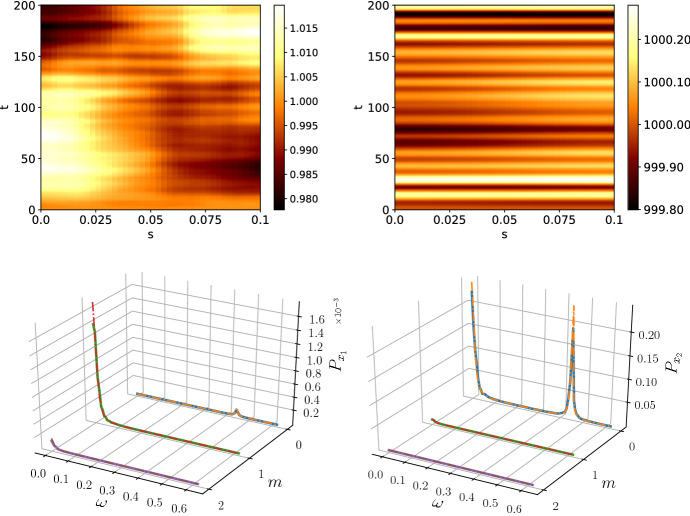
The patterns and power spectra for the species $x_1$ (left) and $x_2$ (right) with noise generated by an Ornstein–Uhlenbeck process and $\tau = 100$. Spatial patterns become visible in the Ornstein–Uhlenbeck case which can be attributed to its dampening effect on temporal oscillations. This can be observed as a visible excitation of the $m=1$ mode in the power spectrum of $x_1$ with the dashed line representing the analytical prediction. The well-known phenomenon of polarity switching (Schumacher et al. 2013) is observed in the pattern of $x_1$. The spectra were averaged over 50 repetitions with $T=1000$ and subsystem size $\varOmega = 100$ (Color figure online)

Hence, it follows that

44 $$\begin{aligned} \langle {\tilde{\eta }}_\kappa (\omega ){\tilde{\eta }}_\kappa (\omega )^\dagger \rangle = B_\kappa \frac{1}{\omega ^2\tau ^2 + 1} \end{aligned}$$

where $B_\kappa$ is the same matrix as in the white noise case and therefore $P(\kappa ,\omega )_\text {Or-Uhl} = P(\kappa ,\omega )_\text {white}/(\omega ^2\tau ^2 +1)$. Hence, the noise colouring will dampen temporal frequencies of $\omega \ne 0$ and, therefore, stabilise the pattern. Consequentially, in systems with Ornstein–Uhlenbeck noise we would expect any present stationary patterns that may be obscured by transient noise effects to be more prominent. We simulated the Schnakenberg system with Ornstein–Uhlenbeck noise and plot the resulting patterns and power spectra in Fig. 3. The pattern of $x_2$ has the same characteristic as in the white noise case, except for a smaller amplitude. Interestingly, $x_1$ shows a very different behaviour to the white noise case. Clear spatial structures can be seen, in particular the phenomenon of polarity switching (Woolley et al. 2011b; Schumacher et al. 2013). A Turing pattern of mode $m=1$ is generated with a given polarity, namely a minimum at $s=0$ and a maximum at $s=L$ or vice versa. Polarity switching describes the inherently stochastic phenomenon of a sudden change in polarity as shown in Fig. 3. The temporal oscillations, although still present, are reduced to a practically unobservable level. We attribute this to the fact that the exponential noise correlations dampen the oscillations present in the white noise system.

### Power Law Noise

By power law noise, we mean a stochastic process whose frequency distribution follows a power law in Fourier space

45 $$\begin{aligned} P_\text {power} = \frac{1}{\omega ^\alpha }. \end{aligned}$$

Various types of power law noise are common in engineering and are defined by colours summarised in Table 2. In this paper, we study red noise, white noise (Sect. 3.2) and violet noise to illustrate the effects of a positive, zero and negative exponent, $\alpha$.

**Table 2:** The various colours of power law noise

Colour	$\alpha$
Red	$\alpha = 2$
Pink	$\alpha =1$
White	$\alpha = 0$
Blue	$\alpha =-1$
Violet	$\alpha = -2$

In this paper, we focus on red and violet noise

**Fig. 4:**
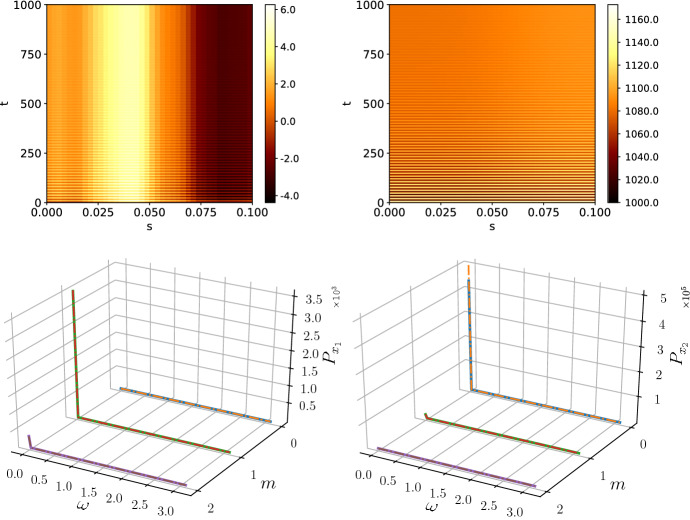
The patterns and power spectra for the species $x_1$ (left) and $x_2$ (right) with noise generated by a red noise process. As predicted, the spectra are dominated by the behaviour the $\omega = 0$, which amounts to the stabilisation of a particular spatial mode. The species concentrations, however, go negative, indicating that a full nonlinear model needs to be used. The peak in the power spectrum for $x_2$ is a numerical artefact. The spectra were averaged over 50 repetitions with simulation time $T=200$. The subsystem size was $\varOmega = 5000$ (Color figure online)

Red noise amplifies small temporal frequencies with $1/\omega ^2 \rightarrow \infty$ as $\omega \rightarrow 0$. Therefore, the zero-frequency behaviour dominates the pattern. As shown in Fig. 4, due to the amplification of small $\omega$, the pattern becomes stable, even though the amplitude grows beyond the biologically viable (non-negative) bound, indicating that nonlinear effects need to be considered in the model. To investigate the red noise further, we look at the noise vector ${\varvec{\eta }}$. For the simulation times chosen, $T=1000$, the noise vector is independent of the time *t* such that each $\eta _i$ has a constant, but random value. Hence, the effect of red noise is deterministic and Eq. (33) is just a linear ordinary differential equation with a constant drift term. After applying the spatial Fourier transform, we obtain the equation

46 $$\begin{aligned} \frac{\hbox {d}{\varvec{\delta x}}_\kappa }{\hbox {d}t} = A_\kappa {\varvec{\delta x}}_\kappa + {\varvec{\eta }}_\kappa , \end{aligned}$$

and denoting the eigenvalues and eigenvectors of $A_\kappa$ as $\lambda _1(\kappa )$, $\lambda _2(\kappa )$ and ${\varvec{\nu }}_1(\kappa )$, ${\varvec{\nu }}_2(\kappa )$, respectively, the linear equation can be solved to give

47 $$\begin{aligned} {\varvec{\delta x}}_\kappa (t) = c_1 {\varvec{\nu }}_1(\kappa ) e^{\lambda _1(\kappa ) t} + c_2{\varvec{\nu }}_2(\kappa ) e^{\lambda _2(\kappa ) t} - A_\kappa ^{-1}{\varvec{\eta }}_\kappa , \end{aligned}$$

where $c_1$ and $c_2$ are constants determined by the initial conditions. Investigating the spatial mode $m = 0$, which corresponds to the dynamics of the non-spatial system, we find that the dynamics corresponds to a stable spiral and, therefore, transient oscillations around $\omega \approx 0.5$ are expected. Indeed, Fig. 4 shows that transient oscillations are present and in order to calculate the power spectrum accurately only the time points in $t\in [800,1000]$ are used. If the entire data were used for the calculation of power spectra, a peak at $\omega \approx 0.5$ would appear which is not accounted for in the analytic spectrum. The same caveat does not apply to other coloured noise processes due to the constantly varying perturbations to the steady state.

**Fig. 5:**
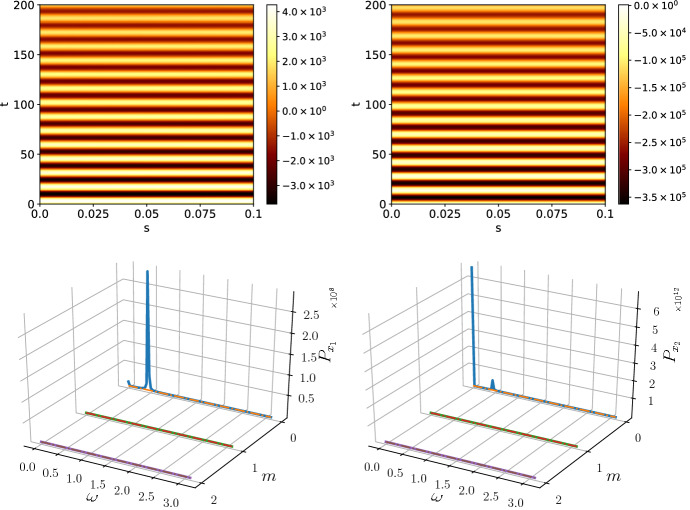
The patterns and power spectra for $x_1$ (left) and $x_2$ (right) with noise generated by a violet noise process. The discrepancy in the power spectra originates from the truncation of the Gaussian process. The negative species concentrations indicate that a nonlinear theory needs to be used to fully model a violet noise system. The spectra were averaged over 50 repetitions with simulation time $T=200$. The subsystem size was $\varOmega = 5000$ (Color figure online)

To illustrate the behaviour of the system on the other end of the colour spectrum, and to highlight the limits of our analysis, we simulated violet noise which stabilised the temporal oscillations further and due to large power at large $\omega$ drove the oscillation amplitude in the linear treatment to unphysical values. Again, this indicates that violet noise cannot be treated in biological applications with a simple linear theory, but a full nonlinear theory must be used. For violet noise, the effect of the truncation of the Gaussian became too large to obtain an accurate prediction of the power spectra as shown in Fig. 5. Further, whilst noise colours with positive exponent are actively studied in biology (Szendro et al. 2001), the potential emergence of blue or violet noise in applications is not clear.

### Stochastic Auxiliary Networks

**Fig. 6:**
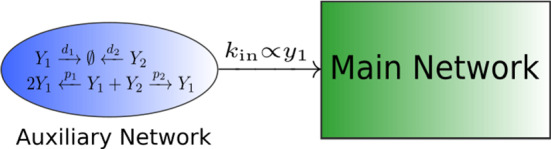
A schematic of an auxiliary network and its input to the main system. This is a specific example of scenario (b) of Fig. 1 (Color figure online)

We now proceed to the second major source of random inflows, namely the dependence on a stochastic auxiliary network. An auxiliary network is a chemical reaction network which provides an input into the Turing system (Fig. 6). The auxiliary network is connected to the main network only via an inflow reaction, and if the auxiliary network reaches a steady state, it can be subsumed into the zero complex for practical modelling. If, however, the auxiliary network exhibits more complex dynamics such as deterministic or stochastic oscillations, then it needs to be treated as a part of the main network. We focus on auxiliary networks which are deterministically stable, but show stochastic quasi-cycles. The dynamics of such systems are modelled by using a correlated stochastic inflow parameter as outlined in Sect. 2.2.

As an illustrative example, we use the predator–prey system described in McKane and Newman (2005),

48 $$\begin{aligned} Y_1\xrightarrow {d_1}&\emptyset \xleftarrow {d_2} Y_2,\nonumber \\ 2Y_1\xleftarrow {p_1}&Y_1+Y_2\xrightarrow {p_2} Y_1. \end{aligned}$$

In McKane and Newman (2005), it was shown that in the deterministic regime system (48) has exactly one attractor for any choice of rate constants. Hence, as the inflow parameters $k_1$ and $k_2$ are proportional to the concentration of $Y_1$, they will have a constant value. However, when the copy numbers of $Y_1$ and $Y_2$ are small and stochastic fluctuations are important, the predator–prey model (48) can exhibit so-called quasi-cycles which are stochastic analogue of limit cycles and manifest themselves as peaks in the power spectra.

Consider this “predator–prey noise” in a Schnakenberg system. Suppose the inflows $k_1$ and $k_2$ in the Schnakenberg system depend on the presence of the chemical species $Y_1$, such that $k_1 \propto y_1$ and $k_2\propto y_1$, where $y_1$ denotes the concentration of $Y_1$. Again, we assume $g_{ij}(t,t') = g(t,t')$ for mathematical simplification. In Fourier space, the power spectrum of $Y_1$ is

49 $$\begin{aligned} P_{\text {Predator}{-}\text {Prey}} = \frac{\alpha + \beta \omega ^2}{\left( \omega ^2+\varOmega ^2_0\right) +\varGamma ^2\omega ^2}, \end{aligned}$$

where we use the parameter values from McKane and Newman (2005) $\alpha = 0.000384,\, \beta = \varGamma = 0.04,\, \varOmega _0^2 = 0.016$.

**Fig. 7:**
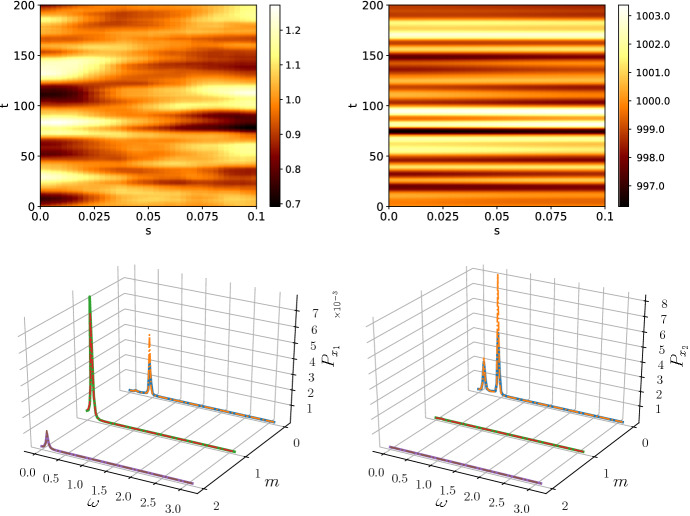
The power spectra for the species $x_1$ (left) and $x_2$ (right) with noise generated by a stochastic predator–prey network (McKane and Newman 2005). The power spectra inherited a second peak from the subsystem which activates oscillatory modes on top of the deterministic oscillation frequency. The spectra were averaged over 50 repetitions and simulation time $T=200$ with *m* indexing the spatial mode. The subsystem size was $\varOmega = 100$ (Color figure online)

Equation (49) has a peak at $\omega \sim 0.06$, so we expect peaks at a similar frequency for each nonzero spatial mode in the power spectrum of the Schnakenberg system. The resulting patterns and the corresponding average power spectra are presented in Fig. 7. The power spectra gained a second peak in the $m=0$ mode and peak in all modes $m>0$ at $\omega \sim 0.06$. Therefore, it can be seen that a deterministic parent system can inherit the dynamics of a stochastic auxiliary network and mix it with its own intrinsic dynamics.

### Mixed Noise

Next, we investigated the case of mixing noise processes; in particular, we consider an Ornstein–Uhlenbeck process which has a different correlation time for $x_1$ and $x_2$, $\tau _1$ and $\tau _2$, respectively. Then, we let $\tau _2 \rightarrow 0$ in order to recover the white noise case for species two.

We introduce the auxiliary normal stochastic processes $\xi$, and hence, the spatial modes of the noise $\eta _\kappa$ are described by Hanggi and Jung (1995)

50 $$\begin{aligned} \frac{\hbox {d}\eta _\kappa }{\hbox {d}t} = -\begin{pmatrix} \tfrac{1}{\tau _1}&{} 0\\ 0 &{} \tfrac{1}{\tau _2} \end{pmatrix}\eta _\kappa + \begin{pmatrix} \tfrac{1}{\tau _1}&{} 0\\ 0 &{} \tfrac{1}{\tau _2} \end{pmatrix}b_\kappa \xi , \end{aligned}$$

where $b_\kappa$ is a $2 \times 4$ matrix satisfying $b_\kappa b_\kappa ^\mathrm{T} = B_\kappa$. We Fourier transform (50) and define the matrix,

51 $$\begin{aligned} \phi = \begin{pmatrix} \tfrac{1}{\tau _1}+i\omega &{} 0\\ 0 &{} \tfrac{1}{\tau _2}+i\omega \end{pmatrix} \end{aligned}$$

to give

52 $$\begin{aligned} {\tilde{\eta }}_\kappa = \phi ^{-1}\begin{pmatrix} \tfrac{1}{\tau _1}&{} 0\\ 0 &{} \tfrac{1}{\tau _2} \end{pmatrix}b_\kappa {\tilde{\xi }}. \end{aligned}$$

Letting $\tau _2\rightarrow 0$ and computing the covariance matrix give

53 $$\begin{aligned} N = \langle {\tilde{\eta }}_\kappa (\omega ){\tilde{\eta }}_\kappa (\omega )^\dagger \rangle = \begin{pmatrix} B_{\kappa ,11}\tfrac{1}{1+\omega ^2\tau _1^2} &{} B_{\kappa ,12}\tfrac{1}{1+i\omega \tau _1}\\ B_{\kappa ,12}\tfrac{1}{1-i\omega \tau _1} &{} B_{\kappa ,22} \end{pmatrix}, \end{aligned}$$

where $B_{k,ij}$ are the *ij*th elements of the $B_\kappa$ matrix. Note that *N* is Hermitian and therefore we expect real power spectra for the patterns of $x_1$ and $x_2$.

**Fig. 8:**
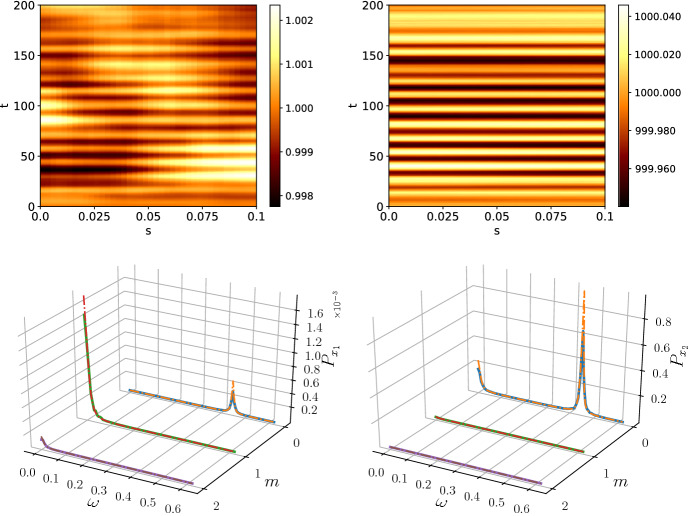
The patterns generated for the species $x_1$ (left) and $x_2$ (right) with Ornstein–Uhlenbeck noise and $\tau = 100$ for $x_1$ and $x_2$ with white noise. The patterns are phenomenologically similar to the Ornstein–Uhlenbeck patterns, however, with reduced amplitude. The power spectrum of $x_2$ is a mixture of white noise and Ornstein–Uhlenbeck noise. The power spectra were averaged over 50 simulations with simulation time $T=1000$ and subsystem size $\varOmega =5000$ (Color figure online)

Simulating Eq. (15) with the noise process described by (50) gives rise to the mixed patterns shown in Fig. 8. Note that computationally the limit $\tau _2\rightarrow 0$ is equal to setting $\tau _2$ to the time step *dt*. The patterns of both species appear similar to the pure Ornstein–Uhlenbeck patterns, however, with reduced amplitude.

### Reduced Stochastic Inflows

In the final subsection, we instigate the case of only species $x_1$ being subjected to stochastic inflows. The species $x_2$ is assumed to only have deterministic inflow at a rate $k_2$ and $\eta _2 = 0$. Hence, the correlation matrices *N* will be reduced to

54 $$\begin{aligned} N = \begin{pmatrix} \langle {\tilde{\eta }}_{1\kappa }{\tilde{\eta }}_{1\kappa }^*\rangle &{} 0\\ 0 &{} 0 \end{pmatrix}. \end{aligned}$$

In the noise processes studied in this paper, the behaviour of the species is virtually unchanged and the patterning is robust with respect to disregarding cross-correlations.

## Conclusion

In this paper, we investigated the effect of stochastic inflows on a deterministic reaction–diffusion system. We restricted the class of networks considered to monostationary systems and used results from real algebraic geometry to show how monostationarity is related to network structure. We then introduced a stochastic perturbation to an inflow reaction as a truncated Gaussian process with the expectation value at the deterministic inflow parameter. After linearising, we computed the power spectra for arbitrary noise colours and showed that in simple cases the power spectra can be derived as a multiplicative factor. The remainder of the paper consisted of applying our analysis to the Schnakenberg system and highlighting the effects various noise colouring can have on a reaction–diffusion system.

We briefly restated the results from the white noise analysis, proceeding to add coloured noise and then demonstrating its effect by computing the power spectra. In particular, for the simple case when all species experience the same temporal correlations, the power spectra of the correlations appear as a multiplicative factor in the total power spectra. Hence, depending on the nature of the correlations they will suppress or excite temporal frequencies at all spatial modes. Ornstein–Uhlenbeck noise has a Lorentzian frequency distribution and, therefore, suppresses positive frequencies. Power law noise can either completely stabilise or destabilise the pattern depending on the sign of the exponent, $\alpha$. This is due to the fact that for positive $\alpha$ temporal frequencies at $\omega = 0$ go to infinity and, therefore, all oscillations are suppressed, which results in a stable pattern which resembles a pattern arising from simulations inside the Turing regime.

For the simulation times used in this paper, the noise vector was actually constant, and therefore, deterministic methods could be used to study the stabilising effect of pink or red noise. The opposite is the case for negative $\alpha$ where the frequency contribution in the power spectrum increases as $\omega$ increases and oscillations are enhanced. However, certain aspects of the red, blue, or violet, noise cases cannot be explained by our simple analytic prediction. The significance and the correct analytic description of blue/violet noise could form a part of further work.

When turning to stochastic auxiliary networks, we see that the reaction–diffusion system can inherit traits of the dynamics of the auxiliary network. In particular, we showed that when the auxiliary network exhibits a predator–prey dynamic with stochastic oscillations, these oscillations can still be observed in the deterministic main network. We concluded the range of applications by considering mixed noise with Ornstein–Uhlenbeck input for species $x_1$ and white noise for species $x_2$. In this case, the system behaves similar to a system with pure Ornstein–Uhlenbeck dynamics and differences can only be found by a power spectral analysis. Finally, we observed in the cases considered that when only one species experiences stochastic inflow the patterns created are, except for potential special cases, similar to the ones when the inflow to both species is randomised. However, the extent to which such results may hold in generality is for further work.

Further directions could also include attempting to relate these studies to potential mechanisms of left–right symmetry breaking amplification in developmental biology, in particular the impact of induced Nodal production on one side of the embryonic node, which is hypothesised to be driven by ciliary fluid flows and also highly error prone (Blum et al. 2014). It is generally asserted that the resulting interactions of the gene products Nodal and Lefty, which are major contenders as Turing morphogens (Müller et al. 2012; Chen and Schier 2002; Schier 2003), amplify this initial error-prone signal to generate robust patterning, driving downstream developmental left–right asymmetry. However, the ability of Turing systems to amplify the spatially localised, error-prone and thus stochastic influx of activator morphogen (Blum et al. 2014, Figure 2) to robustly amplify a stochastic symmetry breaking in self-organisation, as well as any additional constraints required to do so, is theoretically untested. Thus, examining the mechanistic basis of these postulates in this critical developmental biological process provides a fundamental application for the theoretical foundations developed here.

## References

[CR1] Adamer MF, Woolley TE, Harrington HA (2017). Graph-facilitated resonant mode counting in stochastic interaction networks. J R Soc Interface.

[CR2] Alberts B, Johnson A, Lewis J, Raff M, Roberts K, Walter P (2007). Molecular biology of the cell.

[CR3] Banaji M, Pantea C (2018). The inheritance of nondegenerate multistationarity in chemical reaction networks. SIAM J Appl Math.

[CR4] Banerjee K, Bhattacharyya K (2014) Open chemical reaction networks, steady-state loads and Braess-like paradox. ArXiv e-prints

[CR5] Biancalani T, Galla T, McKane AJ (2011). Stochastic waves in a Brusselator model with nonlocal interaction. Phys Rev E.

[CR6] Blum M, Feistel K, Thumberger T, Schweickert A (2014). The evolution and conservation of left-right patterning mechanisms. Development.

[CR7] Briggs W, Henson V (1995). The DFT: an owner’s manual for the discrete Fourier transform.

[CR8] Butler T, Goldenfeld N (2011). Robust ecological pattern formation induced by demographic noise. Phys Rev E.

[CR9] Cartwright JHE (2002). Labyrinthine Turing pattern formation in the cerebral cortex. J Theor Biol.

[CR10] Castets V, Dulos E, Boissonade J, De Kepper P (1990). Experimental evidence of a sustained standing Turing-type nonequilibrium chemical pattern. Phys Rev Lett.

[CR11] Chen Y, Schier AF (2002). Lefty proteins are long-range inhibitors of squint-mediated nodal signaling. Curr Biol.

[CR12] Dauxois T, Di Patti F, Fanelli D, McKane AJ (2009). Enhanced stochastic oscillations in autocatalytic reactions. Phys Rev E.

[CR13] Feinberg M (1987). Chemical reaction network structure and the stability of complex isothermal reactors-I. The deficiency zero and deficiency one theorems. Chem Eng Sci.

[CR14] Feinberg M (1988). Chemical reaction network structure and the stability of complex isothermal reactors-II. Multiple steady states for networks of deficiency one. Chem Eng Sci.

[CR15] Félix B, Shiu A, Woodstock Z (2016). Analyzing multistationarity in chemical reaction networks using the determinant optimization method. Appl Math Comput.

[CR16] Flach EH, Schnell S, Norbury J (2007). Turing pattern outside of the Turing domain. Appl Math Lett.

[CR17] Frank SA (2013). Input–output relations in biological systems: measurement, information and the Hill equation. Biol Direct.

[CR18] García-Ojalvo J, Sancho J (2012). Noise in spatially extended systems.

[CR19] Gillespie DT (1977). Exact stochastic simulation of coupled chemical reactions. J Phys Chem.

[CR20] Gillespie DT (2000). The chemical Langevin equation. J Chem Phys.

[CR21] Hanggi P, Jung P (1995). Colored noise in dynamical systems. Adv Chem Phys.

[CR22] Hochberg D, Lesmes F, Morán F, Pérez-Mercader J (2003). Large-scale emergent properties of an autocatalytic reaction-diffusion model subject to noise. Phys Rev E.

[CR23] Horn F, Jackson R (1972). General mass action kinetics. Arch Ration Mech Anal.

[CR24] Iron D, Wei J, Winter M (2004). Stability analysis of Turing patterns generated by the Schnakenberg model. J Math Biol.

[CR25] Joshi B, Shiu A (2014) A survey of methods for deciding whether a reaction network is multistationary. ArXiv e-prints

[CR26] Kloeden PE, Platen E (1995). Numerical solution of stochastic differential equations.

[CR27] Kondo Sh, Miura T (2010). Reaction-diffusion model as a framework for understanding biological pattern formation. Science.

[CR28] Lenive O, Kirk PDW, Stumpf MPH (2016). Inferring extrinsic noise from single-cell gene expression data using approximate Bayesian computation. BMC Syst Biol.

[CR29] Maini PK, Woolley TE, Baker RE, Gaffney EA, Lee SS (2012). Turing’s model for biological pattern formation and the robustness problem. J R Soc Interface Focus.

[CR30] McKane AJ, Newman TJ (2005). Predator-prey cycles from resonant amplification of demographic stochasticity. Phys Rev Lett.

[CR31] McKane AJ, Biancalani T, Rogers T (2014). Stochastic pattern formation and spontaneous polarisation: the linear noise approximation and beyond. Bull Math Biol.

[CR32] Milshtein GN, Tret ’yakov MV, (1994) Numerical solution of differential equations with colored noise. J Stat Phys 774(3):691–715

[CR33] Müller P, Rogers KW, Jordan BM, Lee JS, Robson D, Ramanathan S, Schier AF (2012). Differential diffusivity of nodal and lefty underlies a reaction-diffusion patterning system. Science.

[CR34] Müller S, Feliu E, Regensburger G, Conradi C, Shiu A, Dickenstein A (2016). Sign conditions for injectivity of generalized polynomial maps with applications to chemical reaction networks and real algebraic geometry. Found Comput Math.

[CR35] Murray JD (2008). Mathematical biology II—spatial models and biomedical applications.

[CR36] Picco N, Sahai E, Maini PK, Anderson ARA (2017). Integrating models to quantify environment mediated drug resistance. Cancer Res.

[CR37] Sagués F, Sancho JM, García-Ojalvo J (2007). Spatiotemporal order out of noise. Rev Mod Phys.

[CR38] Schier AF (2003). Nodal signaling in vertebrate development. Annu Rev Cell Dev Biol.

[CR39] Schnakenberg J (1979). Simple chemical reaction systems with limit cycle behaviour. J Theor Biol.

[CR40] Schumacher LJ, Woolley TE, Baker RE (2013). Noise-induced temporal dynamics in Turing systems. Phys Rev E.

[CR41] Smith GD (1985). Numerical solution of partial differential equations: finite difference methods.

[CR42] Szendro P, Vincze G, Szasz A (2001). Bio-response to white noise excitation. Electro- Magnetobiol.

[CR43] Timmer J, Koenig M (1995). On generating power law noise. Astron Astrophys.

[CR44] Turing A (1952). The chemical basis of morphogenesis. Philos Trans R Soc Lond B Biol Sci.

[CR45] van Kampen NG (1983). Stochastic processes in physics and chemistry.

[CR46] Ward MJ, Wei J (2002). The existence and stability of asymmetric spike patterns for the Schnakenberg model. Stud Appl Math.

[CR47] Woolley TE, Baker RE, Gaffney EA, Maini PK (2011). Influence of stochastic domain growth on pattern nucleation for diffusive systems with internal noise. Phys Rev E.

[CR48] Woolley TE, Baker RE, Gaffney EA, Maini PK (2011). Stochastic reaction and diffusion on growing domains: Understanding the breakdown of robust pattern formation. Phys Rev E.

[CR49] Woolley TE, Baker RE, Gaffney EA, Maini PK (2011). Power spectra methods for a stochastic description of diffusion on deterministically growing domains. Phys Rev E.

[CR50] Woolley TE, Baker RE, Gaffney EA, Maini PK, Seirin-Lee S (2012). Effects of intrinsic stochasticity on delayed reaction-diffusion patterning systems. Phys Rev E.

[CR51] Woolley TE, Baker RE, Maini PK, Cooper S, Soskova M (2017). Turing’s theory of morphogenesis: where we started, where we are and where we want to go. The incomputable.

[CR52] Xavier D (2013) On the theory of cell migration: durotaxis and chemotaxis. Ph.D. thesis, Universitat Politcnica de Catalunya

